# Optical Coherence Tomography Angiography in Age-Related Macular Degeneration

**DOI:** 10.22336/rjo.2026.27

**Published:** 2026

**Authors:** Alexandru-Ionut Duculescu, Ruxandra Pirvulescu, Aida Geamanu, Eylul Firinciogullari, Eduard Toma, Cristina Alexandrescu, Horia Tudor Stanca

**Affiliations:** 1 “Carol Davila” University of Medicine and Pharmacy, Bucharest, Romania; 2 Department of Ophthalmology, University Emergency Hospital, Bucharest, Romania; 3 Department of Ophthalmology, “Prof. Dr. Agrippa Ionescu” Emergency Hospital, Bucharest, Romania

**Keywords:** age-related macular degeneration, optical coherence tomography angiography, macular neovascularization, choriocapillaris, geographic atrophy, AMD = Age-related macular degeneration, CNV = choroidal neovascularization, CVD = choriocapillaris vascular density, GA = geographic atrophy, MNV = macular neovascularization, nAMD = neovascular AMD, OCT = optical coherence tomography, OCTA = optical coherence tomography angiography, ODS = optical coherence tomography-reflective drusen substructures, RPE = retinal pigment epithelium, SD-OCTA = spectral-domain OCTA, SS-OCTA = swept source OCTA, VEGF = vascular endothelial growth factor

## Abstract

**Objective:**

To review and synthesize current evidence regarding the importance of optical coherence tomography angiography (OCTA) in the assessment of choriocapillaris alterations and macular neovascularization (MNV) across the spectrum of age-related macular degeneration (AMD), focusing on disease progression and clinical implications.

**Methods:**

Selective review of the specialized literature, concentrating on both qualitative and quantitative OCTA analyses of the retinal and choroidal microvasculature in AMD. We focused on measures related to choriocapillaris flow deficits, the morphology and maturity of MNV, and their associations with structural changes like drusen, geographic atrophy, and exudative conditions. The review included findings from both spectral-domain and swept-source OCTA systems.

**Results:**

OCTA enables non-invasive, depth-resolved visualization of choriocapillaris and MNV alterations throughout the progression of AMD. In the early and intermediate stages of AMD, localized choriocapillaris flow deficits are often observed beneath drusen and around lesion areas. In more advanced stages, there is significant choriocapillaris damage, especially near geographic atrophy and macular neovascularization. Quantitative OCTA biomarkers, such as density and flow deficit percentage, show strong correlations with disease stage, progression risk, and treatment response. Additionally, the maturity patterns of MNV identified through OCTA seem to affect the integrity of the surrounding choriocapillaris and the dynamics of atrophy.

**Discussion:**

The reviewed studies support a close relationship between choriocapillaris dysfunction, the development of macular neovascularization, and the progression of AMD. These findings reinforce the concept that vascular alterations play a central role throughout the disease spectrum and highlight the potential of OCTA to provide biomarkers that may improve prognostic assessment and treatment monitoring. However, further standardization of imaging protocols and quantitative analysis methods is needed before these biomarkers can be fully integrated into routine clinical practice.

**Conclusions:**

OCTA has become a crucial imaging tool for understanding microvascular changes in AMD. The quantitative evaluation of the choriocapillaris and detailed analysis of MNV morphology offer important insights into disease mechanisms, progression risk, and treatment monitoring. Incorporating OCTA-derived biomarkers into routine clinical practice could improve personalized management strategies for patients across the spectrum of AMD.

## Introduction

Age-related macular degeneration (AMD) is a leading cause of irreversible loss of central vision in industrialized countries. The risk of developing the condition increases after the age of 70 [1]. From a clinical perspective, AMD includes a spectrum ranging from the early and intermediate stages, which are characterized by the presence of drusen and pigmentary abnormalities, to the late stages, which are defined by geographic atrophy or macular neovascularization (MNV), with substantial heterogeneity in terms of both morphology and visual prognosis [2].

From a biological perspective, age-related modifications in the retinal pigment epithelium (RPE), Bruch’s membrane, and the choriocapillaris, in conjunction with oxidative stress, chronic inflammation, and genetic susceptibility, are the primary factors contributing to the development and progression of age-related macular degeneration. Histological and imaging studies have confirmed the important role of the choriocapillaris in the development and progression of this condition. Indeed, early flow impairment and structural rarefaction have been shown to precede overt atrophy or exudation in a significant proportion of affected eyes [3].

Over the past decade, optical coherence tomography angiography (OCTA) has become an important non-invasive imaging tool for evaluating age-related macular degeneration, enabling depth-resolved visualization of the retinal and choroidal microvasculature without the need for exogenous dye administration [4,5]. In dry AMD, optical coherence tomography (OCT) reveals patchy choriocapillaris flow deficits beneath drusen and profound flow loss in areas of geographic atrophy. Conversely, in exudative AMD, OCT provides detailed reconstructions of the microvascular networks and their dynamic response to anti-vascular endothelial growth factor (anti-VEGF) therapy [4-6].

In addition to qualitative visualization, OCTA has stimulated the development of quantitative vascular biomarkers, including vessel density, perfusion density, and choriocapillary flow deficit metrics, which can improve risk stratification for the transition from intermediate to late AMD and enable more accurate assessment of treatment response [5,7]. However, these indicators are influenced by device-specific factors, segmentation strategies, and image processing pipelines, highlighting the need for critical assessment and standardization [7,8].

## Methods

A narrative review of the literature was conducted using the PubMed database to identify English-language studies examining optical coherence tomography angiography (OCTA) findings in age-related macular degeneration. Search terms included “age-related macular degeneration”, “OCTA”, “choriocapillaris”, “macular neovascularization”, “geographic atrophy” and related keywords. The review included studies that focused on both qualitative and quantitative OCTA evaluations of the retinal and choroidal microvasculature across the AMD spectrum, highlighting imaging techniques, vascular biomarkers, and reported clinical correlations.

### Principles of OCT Angiography and Methodological Considerations

OCTA detects blood flow by quantifying temporal variations in the OCT signal induced by the movement of erythrocytes, using amplitude or phase-based decorrelation algorithms applied to repeated B-scans obtained at the same retinal locations [5]. The commercially available OCTA systems in operation today are of two main types: the first operates in the spectral-domain configuration, and the second in the swept-source configuration. These platforms offer the advantages of longer imaging wavelengths and higher acquisition speeds, thereby enhancing choroidal penetration and reducing sensitivity roll-off at increasing depths. This is a particular advantage for imaging the choriocapillaris and the subretinal pigment epithelium in macular neovascularization in cases of age-related macular degeneration [4,5].

Spaide et al. highlighted that OCTA can produce several artifacts that may mimic pathology, including movement and blinking artifacts, projection artifacts from overlying retinal vessels, segmentation errors in eyes with outer retinal distortion, and signal reduction due to media opacity or overlying structures such as drusen [7]. It is important to implement quality control measures and, when possible, manually refine segmentation when interpreting choriocapillaris slabs in AMD cases, as signal attenuation may be misinterpreted as a true flow deficit, especially beneath large drusen or fibrotic scars [7,8].

Quantitative OCTA measurements require standardized acquisition protocols and post-processing procedures. Retinal vessel density measurements are highly repeatable in normal eyes and in cases of age-related macular degeneration when signal strength and segmentation are effectively managed [9]. However, choroidal analyses are more susceptible to variations in thresholding, signal attenuation compensation, and the choice of flow-deficit size filters. These methodological details must be considered when comparing results across studies and when applying research findings to clinical decision-making [5].

### OCTA in Dry (Non-Neovascular) Age-Related Macular Degeneration

In early and intermediate age-related macular degeneration, Waheed et al. have analyzed a heterogeneous pattern of choriocapillaris flow impairment beneath and around drusen on optical coherence tomography angiography, often manifesting as patchy flow deficits interspersed with relatively preserved lobules [4]. Savastano et al. confirmed in their study that these changes reflect both real loss or rarefaction of the choriocapillaris and shadowing from overlying deposits and changes in RPE, but that persistent perfusion deficits persist after correcting for these artifacts in association with AMD lesions [10].

In their study, Veerappan et al. studied optical coherence tomography-reflective drusen substructures (ODS) in intermediate age-related macular degeneration. They demonstrated that specific ODS phenotypes, including low-reflective cores and conical debris, are associated with an elevated risk of progression to geographic atrophy (GA) [1,1]. Similarly, Borrelli et al. reported that regions with high-risk structural biomarkers frequently overlap with regions of reduced choriocapillaris flow on optical coherence tomography angiography, suggesting that local vascular compromise contributes to outer retinal vulnerability [12].

In eyes with geographic atrophy, Choi et al. demonstrated severe loss of choriocapillary flow under the atrophic area and variable degrees of impairment beyond the visible margins using ultrahigh-speed swept-source OCTA [1,3]. Savastano et al. and other researchers have identified an asymmetric border zone of choriocapillaris rarefaction surrounding atrophic lesions, where more severe flow impairments are associated with accelerated lesion growth, highlighting the pivotal role of choriocapillaris dysfunction in the progression of atrophy [10,14].

### OCTA in Exudative (Neovascular) Age-Related Macular Degeneration

In the context of neovascular AMD (nAMD), Kuehlewein et al. pioneered the use of OCTA to characterize type 1 MNV as a tangled sub-RPE network, distinguished by branching and anastomotic patterns that frequently correspond to occult choroidal neovascularization (CNV) on dye angiography [1,5]. Building upon these observations, El Ameen et al. expanded these findings to encompass type 2 MNV, which manifested as a well-delineated subretinal vascular complex overlying the RPE, exhibiting varied en face morphology and growth characteristics compared with type 1 lesions [1,6].

Coscas et al. introduced a qualitative OCTA scoring system for exudative AMD, based on the identification of small branching capillaries, vascular loops, peripheral anastomotic arcades, and a hypointense perilesional “dark halo” in the choriocapillaris [1,7]. Eyes that exhibited several of these characteristics were more prone to show exudation on structural OCT and required continuous anti-VEGF treatment, indicating that OCTA morphology could act as an indicator of neovascular activity [1,7,1,8].

Faatz et al. conducted a quantitative swept-source OCTA analysis of type 1, 2, and 3 MNV, revealing significant disparities in lesion area, fractal dimension, total vessel length, and number of branch nodes among subtypes [1,9]. Type 3 lesions tended to have smaller lesion areas and lower vascular complexity compared with type 1 and 2 lesions, consistent with their origin from the deep retinal capillary plexus and more localized involvement. Conversely, type 1 lesions frequently exhibited extensive horizontal spread beneath the RPE with higher branching indices [1,9,20].

Longitudinal studies using OCTA have shown that MNV undergoes dynamic changes during anti-VEGF treatment. Faatz et al. noted an initial decrease in peripheral capillary sprouting and vascular density, followed by partial reperfusion or maturation in some eyes, even when anatomical fluid had resolved [21]. Cabral et al. proposed that certain baseline OCTA phenotypes, such as a large lesion area and prominent anastomotic arcades, are linked to a higher injection burden and a greater likelihood of recurrence, highlighting the importance of OCTA in tailoring individual treatment plans [2,2].

**Table 1** presents an overview of OCTA features of macular neovascularization reported in the literature.

**Table 1:** OCTA features of macular neovascularization associated with exudative activity and treatment burden in neovascular AMD

Study	MNV subtype/cohort	Key OCTA features	Clinical correlate	Implication
Coscas et al. [**17**]	Type 1-2 MNV, treated exudative AMD	Tiny branching vessels, loops, peripheral arcades, dark halo	Association with exudation on structural OCT	Qualitative OCTA score predicts lesion activity and informs treatment decisions.
Al-Sheikh et al. [**18**]	Mixed MNV subtypes	Vessel density, branching complexity, and anastomoses	Correlation with neovascular activity and visual acuity	OCTA biomarkers may stratify neovascular activity and visual prognosis.
Faatz et al. [**19**]	Type 1, 2, and 3 MNV	Lesion area, fractal dimension, total vessel length, nodes	Distinct vascular signatures by MNV subtype	Supports subtype-specific pathogenesis and may guide personalized monitoring.
Nakano et al. [**20**]	Type 1 vs. type 2 MNV	Vascular maturity indices	Differences in leakage and response to therapy	Type 1 CNVs exhibited lower vessel junction density than type 2 CNVs, suggesting greater vascular maturity.
Cabral et al. [**22**]	Type 1 MNV under long-term anti-VEGF therapy	Immature vs. mature vs. hypermature blood flow patterns	Lower macular atrophy growth rate in sectors with a persistent immature MNV flow pattern compared to mature patterns	Immature Type 1 MNV flow morphology may exert a protective effect against macular atrophy progression

### Non-Exudative Macular Neovascularization and Subclinical Disease

Laiginhas et al. used OCTA to report a high prevalence of non-exudative MNV in eyes with intermediate or advanced non-neovascular AMD, particularly in the fellow eyes of patients with unilateral exudative AMD [23]. The prevalence ranged from 6% to 27%, depending on the specified inclusion criteria. These subclinical type 1 lesions confer an increased risk of subsequent exudation compared with eyes devoid of detectable MNV [2,4].

Roisman et al. demonstrated that non-exudative MNV is typically observed on OCTA as a distinct type 1 network located beneath the RPE. This is often associated with a slight, irregular elevation of the RPE or a “double-layer sign” and may correspond to late indocyanine green angiographic plaques reported in previous studies. These lesions can remain asymptomatic for extended periods and do not show macular fluid or fluorescein leakage, highlighting the unique role of OCTA in their identification [23,2,4].

Treister et al. demonstrated that fellow eyes with subclinical MNV frequently exhibit more extensive choriocapillaris non-perfusion on OCTA than eyes without neovascularization. Furthermore, greater peri-lesional flow deficit burden is associated with a higher risk of conversion to exudative disease [2,5]. These findings suggest that non-exudative MNV and concomitant choriocapillaris impairment represent intermediate stages along a continuum from intermediate AMD to exudative AMD.

### Quantitative OCTA Assessment of the Choriocapillaris Across the AMD Spectrum

Savastano et al. assessed choriocapillaris vascular density (CVD) at various stages of AMD using spectral-domain OCTA. They found that both macular and foveal CVD were notably diminished in cases of late AMD, geographic atrophy, and disciform scars when compared to healthy eyes. In contrast, early AMD exhibited relatively intact macular CVD but decreased foveal CVD. This evidence indicates a progressive, stage-related decline in choriocapillaris perfusion, beginning with the foveal center and extending to broader macular alterations in more advanced stages of the disease [2,6].

In a separate cohort of eyes with advanced exudative age-related macular degeneration that had been treated with multiple anti-vascular endothelial growth factor (VEGF) injections, Savastano et al. demonstrated marked reductions in choriocapillary vascular density in both the whole macula and the foveal regions compared with age-matched healthy controls, independent of the area of neovascularization. This suggests that impairment to the choriocapillaris reflects underlying disease severity rather than simply the size of the neovascular complex and highlights the potential of choroidal vasculopathy as a global biomarker of advanced AMD burden [2,7].

Alagorie et al. conducted a quantitative comparison of choriocapillaris flow deficits in eyes with advanced AMD and healthy eyes, revealing increased area and density of flow deficits in diseased eyes, even after accounting for signal attenuation. In a subsequent analysis, the same group demonstrated a greater peri-lesional flow deficit burden around choroidal neovascular membranes, which was associated with more extensive atrophy and, in some cases, worse visual function. This suggests that choriocapillaris dysfunction and MNV development may represent parallel responses to a shared ischemic insult [2,8].

Moult et al. used swept-source OCTA to evaluate the spatial distribution of choroidal neovascularization-induced impairment of the choriocapillaris, and their findings indicated that flow deficits extend beyond the clinically visible lesion, particularly toward subsequent atrophy expansion [2,9]. Similarly, Nassisi et al. and Rinella et al. demonstrated that the magnitude of perilesional choroidal flow impairment surrounding geographic atrophy predicts the direction and rate of atrophy enlargement, thus positioning choroidal flow deficits as promising prognostic biomarkers in late AMD [14,30].

More recently, Tiosano et al. and Corvi et al. reported that in intermediate AMD without apparent atrophy or exudation, eyes with a higher baseline load or a faster progression in choriocapillary flow deficits are more likely to develop new atrophic lesions or to show rapid expansion of incomplete RPE and outer retinal atrophy [31,32].

**Table 2** presents representative OCTA-derived choriocapillaris metrics reported across different stages of AMD.

**Table 2:** Representative OCTA choriocapillaris metrics across different AMD stages

Study	Population / AMD stage	OCTA device/scan	Key choriocapillaris metric	Main finding
Savastano et al. [**10**]	21 healthy eyes; 84 AMD eyes (early, late, GA, disciform scar)	SD-OCTA, 6×6 mm	Macular and foveal CVD (%)	Macular CVD is reduced in late AMD, GA, and disciform scar vs controls; early AMD with preserved macular but reduced foveal CVD.
Savastano et al. [**26**]	Advanced exudative AMD after multiple anti-VEGF injections vs. healthy eyes	SD-OCTA, 6×6 mm	Whole-macula and foveal CVD (%)	Significant reduction of CVD in advanced exudative AMD vs. controls, independent of MNV area.
Alagorie et al. [**27**]	Advanced AMD (GA and CNV) vs. healthy eyes	SS-OCTA, 6×6 mm	Flow deficit area and density	Greater peripheral macular CC impairment in GA than in CNV
Moult et al. [**29**]	Eyes with CNV secondary to AMD	SS-OCTA, 3×3 and 6×6 mm	Peri-lesional flow deficit percentage	Greater peri-lesional choriocapillaris flow deficit burden is associated with more extensive CNV and atrophy.
Nassisi et al. /Rinella et al. [**14,30**]	Eyes with geographic atrophy	SS-OCTA, 6×6 mm	Perilesional flow deficit metrics	Flow-deficit magnitude at the GA margin predicts the direction and rate of GA enlargement.

## Discussions

The incorporation of OCTA into the management of AMD presents distinct opportunities to enhance disease staging, tailor monitoring intervals, and improve treatment approaches. In cases of dry AMD, choriocapillaris metrics derived from OCTA complement structural OCT markers by revealing the vascular foundation that influences drusen development and atrophy progression. Meanwhile, in neovascular AMD, OCTA offers a comprehensive view of MNV architecture and its changes during anti-VEGF therapy [4,5,19].

From a pathophysiological standpoint, converging evidence from histology, structural OCT, and OCTA supports a hemodynamic model in which early impairment of choriocapillaris perfusion leads to RPE stress, drusen formation, and outer retinal degeneration, with MNV serving as an adaptive, though ultimately maladaptive, response to chronic ischemia [3,2,8,3,3]. This framework, building on concepts initially proposed by Friedman, may help explain the seemingly contradictory observations that subclinical type 1 MNV can both signal exudative conversion and potentially slow the expansion of geographic atrophy in certain eyes [2,3,2,9,3,3].

In clinical settings, OCTA can help assess risk across various situations. For intermediate AMD, significant choriocapillaris flow deficits, high-risk structural indicators like ODS, and early non-exudative MNV signs on OCTA might warrant more frequent monitoring and, eventually, targeted participation in intervention trials to prevent progression to advanced AMD [1,1,2,3,31]. In cases of treated neovascular AMD, initial and ongoing OCTA phenotypes could help identify eyes prone to frequent recurrence or rapid atrophy, potentially guiding personalized treatment schedules and the selection of new therapies targeting angiogenic and inflammatory pathways [1,7,2,2,3,3].

Nonetheless, several challenges currently hinder the broad adoption of OCTA-based biomarkers in standard practice. Differences in acquisition settings, segmentation methods, and image-processing techniques (especially for choriocapillaris analysis) hinder comparability between devices and centers [5,7,10]. Projection artifacts, signal loss, and shadowing from drusen, pigment epithelial detachments, or fibrosis can create false flow deficits, requiring careful interpretation and, ideally, standardized attenuation-compensation methods [7,2,7,34]. Moreover, the cross-sectional design of many existing studies and the relatively short follow-up periods in longitudinal cohorts prevent definitive conclusions about the causal links between specific OCTA features and clinical outcomes [10,2,3,31].

Future research should focus on standardizing OCTA acquisition and analysis protocols, incorporating multimodal imaging and functional testing, and validating OCTA-derived biomarkers in large, prospective cohorts and clinical trials. Machine learning techniques applied to high-dimensional OCT and OCTA datasets may further improve the identification of subtle vascular patterns that predict progression or treatment response, potentially allowing for earlier intervention and more precise, patient-specific management of AMD [2,9,35,36].

## Conclusions

OCTA has become an essential addition to structural OCT and traditional dye angiography for assessing both dry and wet AMD. In non-neovascular disease, OCTA provides insights into choriocapillaris impairment by disease stage and helps identify eyes at high risk for atrophic progression. For neovascular AMD, OCTA allows for detailed analysis of MNV morphology, detection of non-exudative lesions, and monitoring of vascular changes during anti-VEGF treatment. Quantitative metrics of the choriocapillaris obtained from OCTA have potential as prognostic biomarkers across the spectrum of AMD. However, standardization and prospective validation are necessary before they can be fully incorporated into routine clinical practice and regulatory endpoints. As these issues are resolved, OCTA is expected to become increasingly central to the personalized management of AMD.

## References

[1] Fleckenstein M, Keenan TDL, Guymer RH, Chakravarthy U, Schmitz-Valckenberg S, Klaver CC, Wong WT, Chew EY (2021). Age-related macular degeneration. Nat Rev Dis Primers.

[2] Ferris FL 3rd, Wilkinson CP, Bird A, Chakravarthy U, Chew E, Csaky K, Sadda SR, Beckman Initiative for Macular Research Classification Committee (2013). Clinical classification of age-related macular degeneration. Ophthalmology.

[3] Bhutto I, Lutty G (2012). Understanding age-related macular degeneration (AMD): relationships between the photoreceptor/retinal pigment epithelium/Bruch's membrane/choriocapillaris complex. Mol Aspects Med.

[4] Waheed NK, Moult EM, Fujimoto JG, Rosenfeld PJ (2016). Optical Coherence Tomography Angiography of Dry Age-Related Macular Degeneration. Dev Ophthalmol.

[5] Scharf J, Corradetti G, Corvi F, Sadda S, Sarraf D (2021). Optical Coherence Tomography Angiography of the Choriocapillaris in Age-Related Macular Degeneration. J Clin Med.

[6] Roisman L, Goldhardt R (2017). OCT Angiography: An Upcoming Non-invasive Tool for Diagnosis of Age-related Macular Degeneration. Curr Ophthalmol Rep.

[7] Spaide RF, Fujimoto JG, Waheed NK (2015). Image artifacts in optical coherence tomography angiography. Retina.

[8] Spaide RF, Curcio CA (2017). Evaluation of Segmentation of the Superficial and Deep Vascular Layers of the Retina by Optical Coherence Tomography Angiography Instruments in Normal Eyes. JAMA Ophthalmol.

[9] Lee SC, Tran S, Amin A, Morse LS, Moshiri A, Park SS, Yiu G (2020). Retinal Vessel Density in Exudative and Nonexudative Age-Related Macular Degeneration on Optical Coherence Tomography Angiography. Am J Ophthalmol.

[10] Savastano MC, Fossataro C, Carlà MM, Fantozzi C, Falsini B, Savastano A, Rizzo C, Kilian R, Rizzo S (2022). OCT angiography analysis of choriocapillaris vascular density in different stages of age-related macular degeneration. Front Ophthalmol (Lausanne).

[11] Veerappan M, El-Hage-Sleiman AM, Tai V, Chiu SJ, Winter KP, Stinnett SS, Hwang TS, Hubbard GB, Michelson M, Gunther R, Wong WT, Chew EY, Toth CA, Age-related Eye Disease Study 2 Ancillary Spectral Domain Optical Coherence Tomography Study Group (2016). Optical Coherence Tomography Reflective Drusen Substructures Predict Progression to Geographic Atrophy in Age-related Macular Degeneration. Ophthalmology.

[12] Borrelli E, Uji A, Sarraf D, Sadda SR (2017). Alterations in the Choriocapillaris in Intermediate Age-Related Macular Degeneration. Invest Ophthalmol Vis Sci.

[13] Choi W, Moult EM, Waheed NK, Adhi M, Lee B, Lu CD, de Carlo TE, Jayaraman V, Rosenfeld PJ, Duker JS, Fujimoto JG (2015). Ultrahigh-Speed, Swept-Source Optical Coherence Tomography Angiography in Nonexudative Age-Related Macular Degeneration with Geographic Atrophy. Ophthalmology.

[14] Nassisi M, Baghdasaryan E, Borrelli E, Ip M, Sadda SR (2019). Choriocapillaris flow impairment surrounding geographic atrophy correlates with disease progression. PLoS One.

[15] Kuehlewein L, Bansal M, Lenis TL, Iafe NA, Sadda SR, Bonini Filho MA, De Carlo TE, Waheed NK, Duker JS, Sarraf D (2015). Optical Coherence Tomography Angiography of Type 1 Neovascularization in Age-Related Macular Degeneration. Am J Ophthalmol.

[16] El Ameen A, Cohen SY, Semoun O, Miere A, Srour M, Quaranta-El Maftouhi M, Oubraham H, Blanco-Garavito R, Querques G, Souied EH (2015). Type 2 neovascularization secondary to age-related macular degeneration imaged by optical coherence tomography angiography. Retina.

[17] Coscas F, Lupidi M, Boulet JF, Sellam A, Cabral D, Serra R, Français C, Souied EH, Coscas G (2019). Optical coherence tomography angiography in exudative age-related macular degeneration: a predictive model for treatment decisions. Br J Ophthalmol.

[18] Al-Sheikh M, Iafe NA, Phasukkijwatana N, Sadda SR, Sarraf D (2018). Biomarkers of neovascular activity in age-related macular degeneration using optical coherence tomography angiography. Retina.

[19] Faatz H, Rothaus K, Ziegler M, Book M, Heimes-Bussmann B, Pauleikhoff D, Lommatzsch A (2022). Vascular Analysis of Type 1, 2, and 3 Macular Neovascularization in Age-Related Macular Degeneration Using Swept-Source Optical Coherence Tomography Angiography Shows New Insights into Differences of Pathologic Vasculature and May Lead to a More Personalized Understanding. Biomedicines.

[20] Nakano Y, Kataoka K, Takeuchi J, Fujita A, Kaneko H, Shimizu H, Ito Y, Terasaki H (2019). Vascular maturity of type 1 and type 2 choroidal neovascularization evaluated by optical coherence tomography angiography. PLoS One.

[21] Faatz H, Farecki ML, Rothaus K, Gutfleisch M, Pauleikhoff D, Lommatzsch A (2019). Changes in the OCT angiographic appearance of type 1 and type 2 CNV in exudative AMD during anti-VEGF treatment. BMJ Open Ophthalmol.

[22] Cabral D, Coscas F, Pereira T, Laiginhas R, Rodrigues C, Français C, Nogueira V, Falcão M, Miere A, Lupidi M, Coscas G, Souied E (2021). Implications of the morphologic patterns of type 1 macular neovascularization on macular atrophy growth in patients under anti-vascular endothelial growth factor treatment. Retina.

[23] Laiginhas R, Yang J, Rosenfeld PJ, Falcão M (2020). Nonexudative Macular Neovascularization-A Systematic Review of Prevalence, Natural History, and Recent Insights from OCT Angiography. Ophthalmol Retina.

[24] Roisman L, Zhang Q, Wang RK, Gregori G, Zhang A, Chen CL, Durbin MK, An L, Stetson PF, Robbins G, Miller A, Zheng F, Rosenfeld PJ (2016). Optical Coherence Tomography Angiography of Asymptomatic Neovascularization in Intermediate Age-Related Macular Degeneration. Ophthalmology.

[25] Treister AD, Nesper PL, Fayed AE, Gill MK, Mirza RG, Fawzi AA (2018). Prevalence of Subclinical CNV and Choriocapillaris Nonperfusion in Fellow Eyes of Unilateral Exudative AMD on OCT Angiography. Transl Vis Sci Technol.

[26] Savastano MC, Rizzo C, Gambini G, Savastano A, Falsini B, Bacherini D (2021). Choriocapillaris vascular density changes: healthy vs advanced exudative age-related macular degeneration previously treated with multiple anti-VEGF intravitreal injections. Diagnostics (Basel).

[27] Alagorie AR, Verma A, Nassisi M, Sadda SR (2019). Quantitative Assessment of Choriocapillaris Flow Deficits in Eyes with Advanced Age-Related Macular Degeneration Versus Healthy Eyes. Am J Ophthalmol.

[28] Alagorie AR, Verma A, Nassisi M, Nittala M, Velaga S, Tiosano L, Sadda SR (2020). Quantitative assessment of choriocapillaris flow deficits surrounding choroidal neovascular membranes. Retina.

[29] Moult EM, Alibhai AY, Rebhun C, Lee B, Ploner S, Schottenhamml J, Husvogt L, Baumal CR, Witkin AJ, Maier A, Duker JS, Rosenfeld PJ, Waheed NK, Fujimoto JG (2020). Spatial distribution of choriocapillaris impairment in eyes with choroidal neovascularization secondary to age-related macular degeneration: A Quantitative OCT Angiography Study. Retina.

[30] Rinella NT, Zhou H, Zhang Q, Keiner C, Oldenburg CE, Duncan JL, Wang RK, Schwartz DM (2019). Quantifying Choriocapillaris Flow Voids in Patients With Geographic Atrophy Using Swept-Source OCT Angiography. Ophthalmic Surg Lasers Imaging Retina.

[31] Tiosano L, Corradetti G, Sadda SR (2021). Progression of choriocapillaris flow deficits in clinically stable intermediate age-related macular degeneration. Eye (Lond).

[32] Corvi F, Tiosano L, Corradetti G, Nittala MG, Lindenberg S, Alagorie AR, McLaughlin JA, Lee TK, Sadda SR (2021). Choriocapillaris flow deficits as a risk factor for progression of age-related macular degeneration. Retina.

[33] Friedman E (1997). A hemodynamic model of the pathogenesis of age-related macular degeneration. Am J Ophthalmol.

[34] Borrelli E, Sadda SR, Uji A, Querques G (2019). Pearls and Pitfalls of Optical Coherence Tomography Angiography Imaging: A Review. Ophthalmol Ther.

[35] Jia Y, Bailey ST, Hwang TS, McClintic SM, Gao SS, Pennesi ME, Flaxel CJ, Lauer AK, Wilson DJ, Hornegger J, Fujimoto JG, Huang D (2015). Quantitative optical coherence tomography angiography of vascular abnormalities in the living human eye. Proc Natl Acad Sci U S A.

[36] Schmidt-Erfurth U, Chong V, Loewenstein A, Larsen M, Souied E, Schlingemann R, Eldem B, Monés J, Richard G, Bandello F, European Society of Retina Specialists (2014). Guidelines for the management of neovascular age-related macular degeneration by the European Society of Retina Specialists (EURETINA). Br J Ophthalmol.

